# Odontoid incidence: a constant cervical anatomical feature evident in standing plain radiographs and supine magnetic resonance images

**DOI:** 10.1186/s13018-024-04542-0

**Published:** 2024-01-13

**Authors:** Longao Huang, Weiyou Chen, Hongyuan Xu, Hongyu Qin, Hua Jiang

**Affiliations:** Department of Spine Surgery, The First Affiliated Hospital of Guangxi Medical University, 6 Shuangyong Road, Nanning, 530021 Guangxi Zhuang Autonomous Region China

**Keywords:** Cervical sagittal parameters, Odontoid incidence, Odontoid tilt, C2 slope, CL prediction

## Abstract

**Objective:**

To assess whether there is a difference between measurements of odontoid incidence (OI) and other cervical sagittal parameters by X-ray radiography and those by supine magnetic resonance imaging (MRI).

**Methods:**

Standing X-ray and supine MRI images of 42 healthy subjects were retrospectively analyzed. Surgimap software was employed to measure cervical sagittal parameters including OI, odontoid tilt (OT), C2 slope (C2S), C0-2 angle, C2-7 angle, T1 slope (T1S) and T1S-cervical lordosis (CL). Paired samples t-test was applied to determine the difference between parameters measured by standing X-ray and those by supine MRI. In addition, the statistical correlation between the parameters were compared. The prediction of CL was performed and validated using the formula CL = 0.36 × OI − 0.67 × OT − 0.69 × T1S.

**Results:**

Significant correlations and differences were found between cervical sagittal parameters determined by X-ray and those by MRI. OI was verified to be a constant anatomic parameter and the formula CL = 0.36 × OI − 0.67 × OT − 0.69 × T1S can be used to predict CL in cervical sagittal parameters.

**Conclusions:**

OI is verified as a constant anatomic parameter, demonstrating the necessity of a combined assessment of cervical sagittal balance by using standing X-ray and supine MRI. The formula CL = 0.36 × OI − 0.67 × OT − 0.69 × T1S can be applied to predict CL in cervical sagittal parameters.

## Introduction

The incidence rate of cervical spondylosis increases steadily along with changes in lifestyle and population aging. Its main etiology is cervical disk herniation or foraminal stenosis, which causes radiating pain and numbness in neck, shoulder, and upper extremity. They can lead to long-term disability, pain, and financial burden, and contribute to poor quality of life [1, 2].

Since the sagittal balance was reported to be significantly related to patients’ health-related quality of life (HRQOL), the importance of sagittal alignment and sagittal balance has been gradually recognized [3]. Cervical sagittal imbalance is one of the main reasons for cervical disk degeneration and associated disorders [4–6]. Cervical sagittal balance-related parameters, including C0-2 angle (C0-2), C2–C7 cervical lordosis (CL), C2 slope (C2S), T1 slope (T1S), and T1S minus CL (T1S-CL) [7], are being used to evaluate the severity and treatment outcomes of the disease [8]. Odontoid incidence (OI) is one of the most important anatomic parameters in terms of cervical sagittal balance and equals the sum of the positional parameters odontoid tilt (OT) and C2S (OI = OT + C2S) [9]. OI strongly correlates with CL through C2S, thus largely determining the different cervical types and consequent mechanisms of cervical spine degeneration [9]. The analysis of OI is essential to understand the impact of cervical sagittal alignment and to make an surgical strategy for cervical deformity correction. OT indicates the spatial orientation of the odontoid process, which may vary depending on the balance of cranial and horizontal gaze, and could aid in two-dimensional analyzes of cervical alignment and balance [10].

Different imaging modalities such as standing X-ray radiography [10] and supine magnetic resonance imaging (MRI) [11] are commonly used to assess cervical spine alignment. However, body posture and the role of the head in cervical load-bearing may have an impact on the spine, leading to different results [12–14]. Standing X-ray provides gravity balance information; however, in patients with a short neck or high sternum, the endplates of the lower cervical and upper thoracic vertebrae may not be clear, thus affecting the measurement of cervical sagittal parameters [15]. Supine MRI can provide anatomical structure information of soft tissues with high spatial resolution [16]; however, it cannot reflect the natural upright state of the spine, since cervical balance is obtained in an upright posture. Therefore, to provide a more comprehensive and reliable reference for the prevention, diagnosis and treatment of cervical spine diseases, further studies investigating correlations and differences between cervical spine parameters measured by different imaging methods are needed. In this study, we assessed whether there was a difference between cervical sagittal parameters measured by standing X-ray and those by supine MRI and validated whether OI is a constant anatomic parameter. We explored correlations between cervical sagittal parameters and determined the formula for predicting cervical sagittal parameters.

## Materials and methods

### Case selection

Ethical approval was obtained from the Ethics Committee of our hospital. Adults aged between 18 and 40 aged without spinal symptoms were included in this study. All participants were selected from those population undergoing annual routine health checkups at the Center of Health Management, affiliated with The First Affiliated Hospital of Guangxi Medical University from January 1, 2011 to October 31, 2022.

### Patient selection criteria

The following screening criteria were used for ensuring the health status of study participants and excluding factors that might interfere with results in order to improve the accuracy and reliability of the study.

All participants had to provide available clinical data, including standardized lateral cervical spine radiographs and MRI images to meet the inclusion criteria. Exclusion criteria included a history of cervical spine trauma, infection, tumor, deformity, history of cervical spine surgery, or history of other spinal trauma and disease to avoid measurement bias and disease progression. Additionally, individuals with diagnosed diseases, degenerative changes (.g.,, decreased disk height or osteophyte formation), a history of treatment related to the cervical spine, a history of spinal surgery, or overall sagittal alignment abnormalities were excluded.

### Acquisition conditions

The radiographic protocol was standardized. For each subject, cervical spine lateral radiographs were obtained with a 10 × 12-inch cassette at a 72-inch (182 cm) distance with the radiographic tube centered at the C4–C5 disk space with no magnification. Subjects were instructed to stand in a comfortable position and keep their eyes forward with their arms extended on their chests. The MRI images were obtained using a 1.5-T system (Signa; GE Medical Systems). Subjects were placed in the scanner chamber in a supine position.The cervical cord was imaged in neutral position with a standard MR receive coil (HD Head Neck and Spine Array) dedicated to spinal imaging. The T2-weighted MR images were acquired with a field of view (FOV) of 260 mm and a matrix size of 512 × 512 pixels. Later, MRI data was measured on T2-weighted MR images. The interval between X-ray and MRI examinations was not more than 2 months in each case to avoid the implications of disease progression for the results.

### Data collection and measurement

**Fig. 1:**
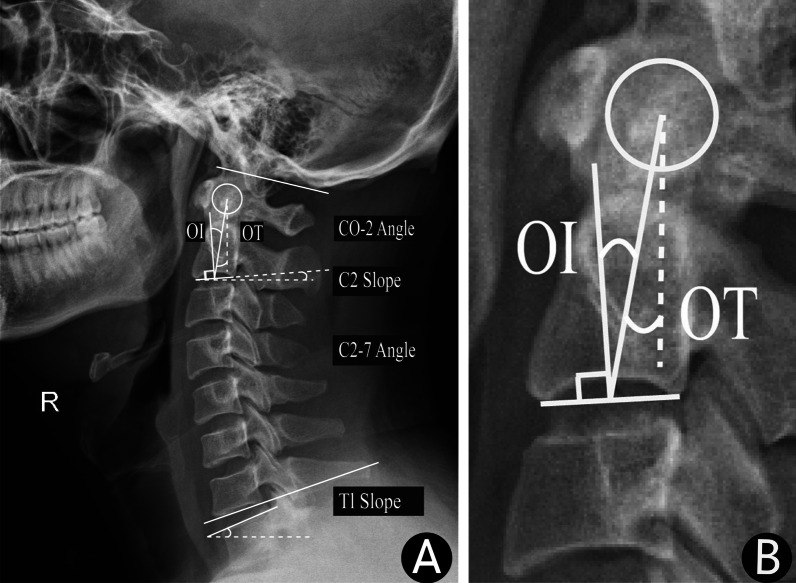
Measurement of odontoid process parameters and cervical spine parameters on DR images. **A**: Measurement of OI, OT and cervical spine parameters on DR images of a healthy 35-year-old male; **B**: High-resolution views of OI and OT on DR images of a healthy 35-year-old male.DR, digital radiography; OI, odontoid incidence; OT, odontoid tilt

**Fig. 2:**
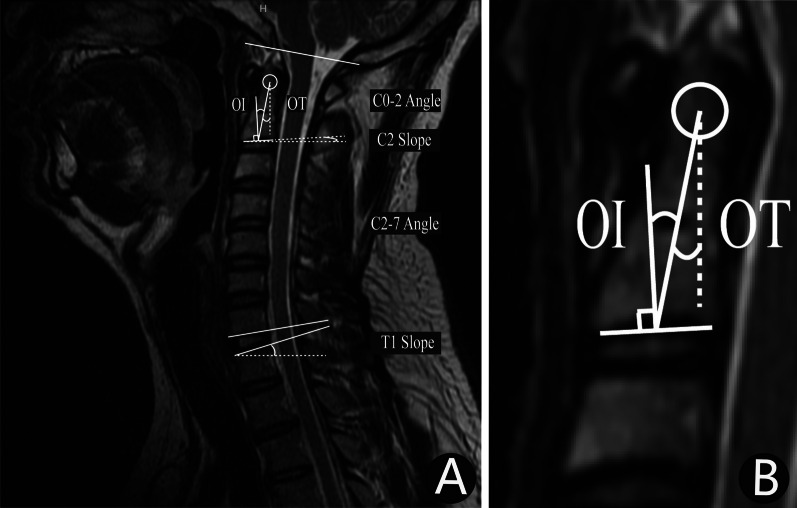
Measurement of odontoid process parameters and cervical spine parameters on MRI images. **A**: Measurement of OI, OT and cervical spine parameters on MRI images of a healthy 35-year-old male; **B**: High-resolution views of OI and OT on MRI images of a healthy 35-year-old male.MRI, magnetic resonance imaging; *OI*, odontoid incidence; *OT*, odontoid tilt

Data was collected by one of investigators in the research team, who would not be involved in final data analysis. The assessment was performed by two senior spine surgeons (with 12 and 6 years' clinical experience, respectively). They performed the following measurements of cervical sagittal parameters on X-ray and MRI images using the Surgimap software (Nemaris, Inc., New York, NY, USA): OI, OT, C2S, C0-2 angle, C2-7 angle, T1S and T1S-CL. OI was defined as the angle between the line perpendicular to the C2 endplate (C2EP) at its midpoint and the line connecting this point to the center of the odontoid process. OT was defined as the angle created by a line running from the C2EP midpoint to the center of the odontoid process and the vertical axis. C2S was defined as the angle between the C2EP and a horizontal line. The C0-2 angle, an angle between the C2EP and the McRae line was measured. The C2-7 angle was measured as the angle between C2 and C7 lower endplates. T1S was defined as an angle formed between the T1 upper endplate and the horizontal plane (Figs. 1, 2). Two assessors completed the measurements independently and averaged them for the final results.

### Statistical analyzes

All data in this study was analyzed by using SPSS (version 26), R (version 4.2.2) and RStudio (version 1.1.463). Inter-observer agreement for each parameter was assessed by the intraclass correlation coefficient (ICC) [17], whose values are expressed as 95% confidence intervals (CI). The ICC takes values ranging from 0 to 1, with a larger value indicating a better agreement. According to the recommendations of Fleiss [18] and Landis [19], ICC values between 0.00 and 0.40 indicate poor agreement, between 0.40 and 0.74 indicate good agreement, and between 0.75 and 1.00 indicate excellent agreement.

In this study, all parameters are presented as mean ± standard deviation. If the parameters conformed to normal distributions, the paired t-test would be carried out to analyze the differences between X-ray and MRI. For correlation analysis between two imaging studies, the Pearson correlation test was used, which was expressed as *r* coefficient. The *r* coefficients ranging from − 1.0 to − 0.5 or 0.5 to 1.0 suggest strong correlation, − 0.5 to − 0.3 or 0.3 to 0.5 indicate moderate correlation, − 0.3 to − 0.1 or 0.1 to 0.3 represent weak correlation, and − 0.1 to 0.1 imply no correlation or very weak correlation. A P value < 0.05 was considered to be statistically significant.

## Results

A total of 96 individuals who participated in health checkups were screened in this study, from whom, 54 were excluded based on the exclusion criteria. The study population consisted of 42 participants [15 (35.7%) males and 27 (64.3%) females] with a mean age of 29.1 ± 5.97 (18–39 years old). The study involved the measurement of seven sagittal parameters and a total of 588 measurements were obtained. Two evaluators performed separately the measurements for the radiographic and MRI studies.

### Inter-observer agreement

By the credibility of analyzes, the inter-observer agreement of all cervical spine parameters on X-ray and MRI was as follows: X-ray: 0.552 (OI), 0.855 (OT), 0.907 (C2S), 0.942 (C0-2), 0.965 (C2-7), 0.952 (T1S), 0.897 (T1S-CL); MRI: 0.723 (OI), 0.819 (OT), 0.958 (C2S), 0.985 (C0-2), 0.963 (C2-7), 0.948 (T1S), 0.924 (T1S-CL). Except for the inter-observer agreement of 0.552 and 0.723 for OI on X-ray and MRI, respectively, both of which were in good agreement, the remaining parameters showed excellent agreement. This indicates that the overall inter-observer agreement was great and the results were statistically significant (P < 0.001) (Table 1).

**Table 1:** Inter-observer reliability and pairwise difference of each parameter between observers

	Observer		Inter-observer reliability	
	A^a^	B^a^		
Parameters	Mean (SD)	Mean (SD)	ICC	*p*
*DR*				
OI (°)	15.37 (2.35)	15.4 (3.18)	0.552	0.007
OT (°)	13.98 (6.27)	11.03 (6.68)	0.855	< 0.001
C2S (°)	1.24 (6.27)	4.37 (6.68)	0.907	< 0.001
C0-2 (°)	22.87 (9.72)	26.34 (9.47)	0.942	< 0.001
C2-7 (°)	21.45 (9.94)	20.07 (9.81)	0.965	< 0.001
T1S (°)	31 (8.13)	32.41 (9.00)	0.952	< 0.001
T1S-CL (°)	9.55 (7.92)	12.35 (9.10)	0.897	< 0.001
*MRI*				
OI (°)	15.9 (4.87)	17.45 (4.86)	0.723	< 0.001
OT (°)	5.10 (7.01)	5.78 (7.14)	0.819	< 0.001
C2S (°)	11.04 (7.02)	11.76 (6.90)	0.958	< 0.001
C0-2 (°)	15.27 (6.28)	14.8 (6.32)	0.985	< 0.001
C2-7 (°)	12.66 (10.28)	11.81 (10.07)	0.963	< 0.001
T1S (°)	28.85 (7.13)	27.89 (7.62)	0.948	< 0.001
T1S-CL (°)	16.19 (8.85)	16.08 (8.09)	0.924	< 0.001

^a^*A*, *B* represent the two observers who participated in the study

*DR* digital radiography; *MRI* magnetic resonance imaging; *OI* odontoid incidence; *OT* odontoid tilt; *C2S* C2 slope; *TIS* T1 slope; *CL* cervical lordosis

### Cervical sagittal parameters

The mean cervical sagittal parameters on X-ray were 15.38° ± 2.32° (OI), 12.51° ± 6.18° (OT), 2.8° ± 6.36° (C2S), 24.6° ± 9.47° (C0-2), 20.76° ± 9.73° (C2-7), 31.71° ± 8.4° (T1S), 10.95° ± 8.21° (T1S-CL). The mean cervical sagittal parameters on MRI were 16.68° ± 4.33° (OI), 5.44° ± 6.51° (OT), 11.4° ± 6.83° (C2S), 15.03° ± 6.26° (C0-2), 12.24° ± 10° (C2-7), 28.37° ± 7.21° (T1S), 16.13° ± 8.17° (T1S-CL). After X-ray and MRI paired t-test, the results were: − 1.29° ± 4.38° (OI), 7.07° ± 7.39° (OT), − 8.6° ± 7.03° (C2S), 9.57° ± 9.68° (C0-2), 8.52° ± 9.59° (C2-7), 3.34° ± 7.42° (T1S), − 5.19° ± 7.95° (T1S-CL). The results showed that there was no significant difference of OI in X-ray and MRI (*p* > 0.05), and OT, C0-2, C2-7, and T1S were significantly greater on X-ray than on MRI (*p* < 0.05), while C2S and T1S-CL were significantly smaller on X-ray than on MRI (*p* < 0.05) (Table 2).

**Table 2:** Pairwise differences of cervical sagittal parameters between DR and MRI

	DR	MRI	Pairwise Difference	
Parameter	Mean (SD)	Mean (SD)	Mean (SD)	*p* value
OI (°)	15.38 (2.32)	16.68 (4.33)	− 1.29 (4.38)	0.063
OT (°)	12.51 (6.18)	5.44 (6.51)	7.07 (7.39)	< 0.001
C2S (°)	2.80 (6.36)	11.40 (6.83)	− 8.60 (7.03)	< 0.001
C0-2 (°)	24.60 (9.47)	15.03 (6.26)	9.57 (9.68)	< 0.001
C2-7 (°)	20.76 (9.73)	12.24 (10)	8.52 (9.59)	< 0.001
T1S (°)	31.71 (8.40)	28.37 (7.21)	3.34 (7.42)	0.006
T1S-CL (°)	10.95 (8.21)	16.13 (8.17)	− 5.19 (7.95)	< 0.001

*DR* digital radiography; *MRI* magnetic resonance imaging; *OI* odontoid incidence; *OT* odontoid tilt; *C2S* C2 slope; *TIS* T1 slope; *CL* cervical lordosis

### X-ray and MRI parameter correlation

After Pearson's analysis, the same cervical sagittal parameters on X-ray and MRI were found to be significantly correlated (*p* < 0.05), including C2-7 (*r* = 0.528), T1S (*r* = 0.557), T1S-CL (*r* = 0.529), C2S (*r* = 0.433), and OT (*r* = 0.322). Whereas, C0-2 and OI did not have a significant correlation between X-ray and MRI (*p* > 0.05).

#### Parameter correlations

**Fig. 3:**
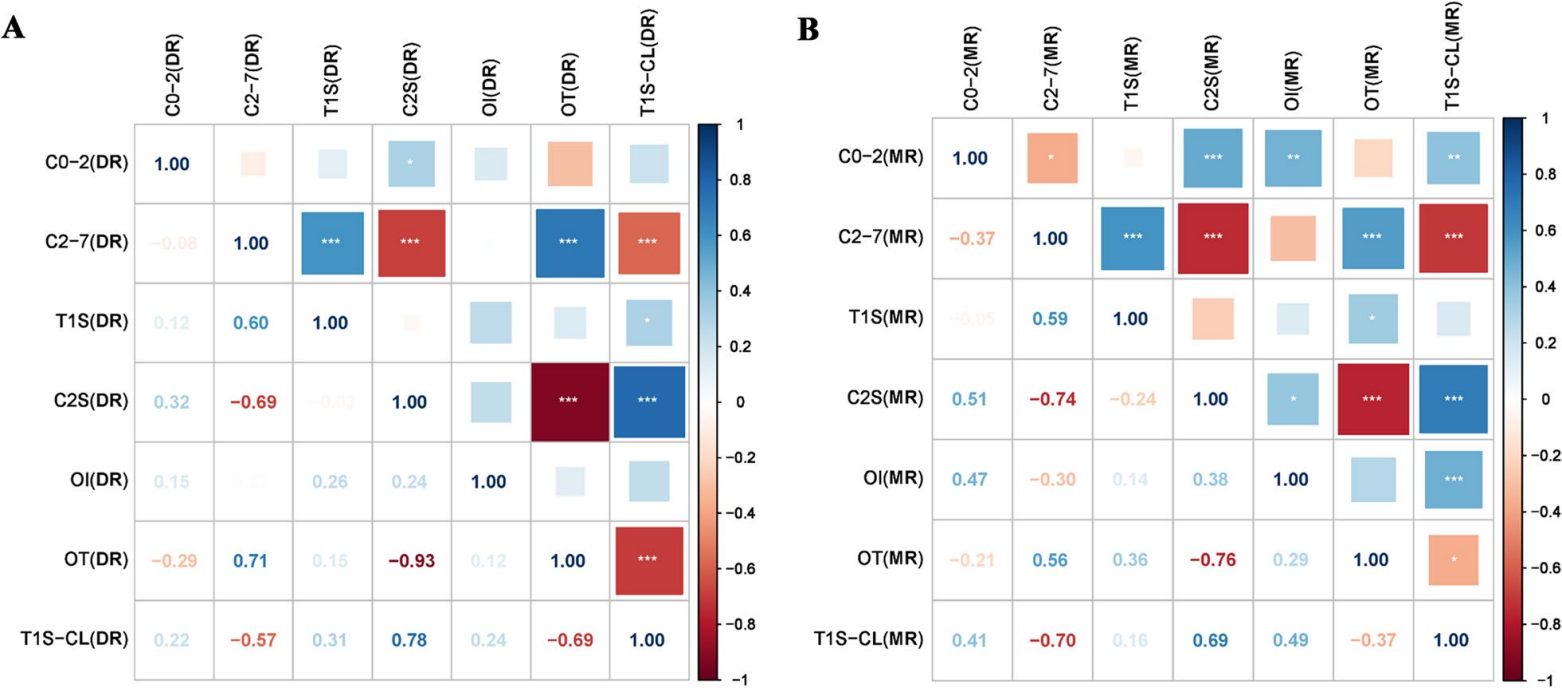
Heat map of correlations. **A**: Pearson correlation analysis between OI, OT and cervical parameters on cervical DR images; **B**: Pearson correlation analysis between OI, OT and cervical parameters on cervical MRI images. *OI* odontoid incidence; *OT* odontoid tilt; *DR* digital radiography; *MRI* magnetic resonance imaging

After Pearson's analysis, the correlations between X-ray cervical spine parameters were as follows: OT was significantly correlated with C2S (*r* = − 0.93), C2-7 (*r* = 0.71), and T1S-CL (*r* = − 0.69), but not with OI, C0-2, and T1S. C2S was significantly correlated with C0-2 (*r* = 0.32), C2-7 (*r* = -0.69), and T1S-CL (*r* = 0.78). There was no significant correlation between OI and the other parameters. C2-7 was significantly correlated with C2S (*r* = − 0.69), OT (*r* = 0.71) and T1S-CL (*r* = − 0.57). T1S was significantly correlated with C2-7 (*r* = 0.6) and T1S-CL (*r* = 0.31). T1S-CL was significantly correlated with C2S (*r* = 0.78), C2-7 (*r* = − 0.57), OT (*r* = − 0.69) and T1S (*r* = 0.31) (Fig. 3).

For the MRI data, we derived the following correlation results: The OT was significantly correlated with C2S (*r* = − 0.76), C2-7 (*r* = 0.56), T1S (*r* = 0.36), and T1S-CL (*r* = − 0.37), but not with OI and C0-2. C2S was significantly correlated with C0-2 (*r* = 0.51), C2-7 (*r* = − 0.74), OI (*r* = 0.38), OT (*r* = − 0.76), and T1S-CL (*r* = 0.69), but not with T1S. OI was significantly correlated with C2S (*r* = 0.38), C0-2 (*r* = 0.47), and T1S-CL (*r* = 0.49), but not with OT, C2-7, and T1S. C2-7 was significantly correlated with C2S (*r* = − 0.74), OT (*r* = 0.56), C0-2 (*r* = − 0.37), T1S (*r* = 0.59), and T1S-CL (*r* = − 0.7), but not with OI. T1S was significantly correlated with C2-7 (*r* = 0.59) and OT (*r* = 0.36). T1S-CL was significantly correlated with C0-2 (*r* = 0.41), OI (*r* = 0.49), C2S (*r* = 0.78), C2-7 (*r* = − 0.7) and OT (*r* = − 0.37), but not with T1S (Fig. 3).

### Validation of formula efficacy

**Fig. 4:**
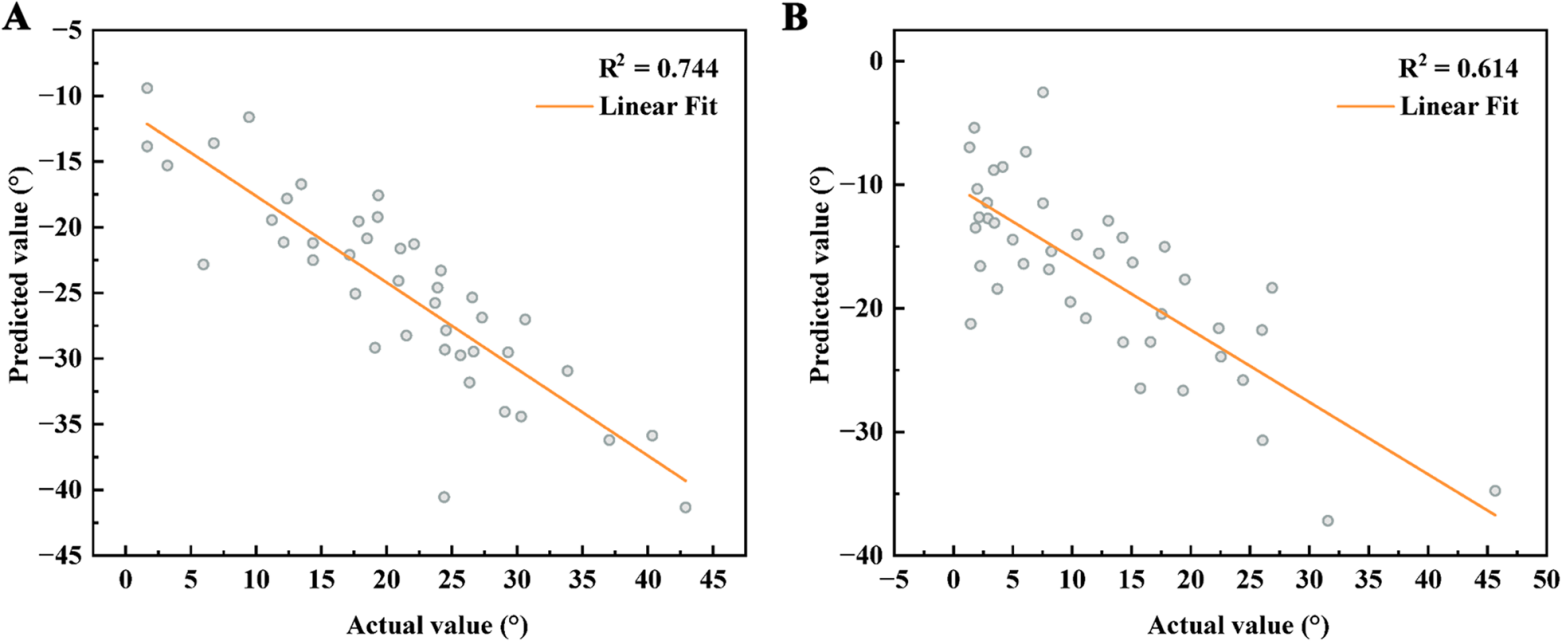
Validation of CL prediction formula efficacy. **A**: Validation of CL prediction formula efficacy on DR images; **B**: Validation of CL prediction formula efficacy on MRI images. R2 represents the coefficient of determination. *CL* cervical lordosis; *DR* digital radiography; *MRI* magnetic resonance imaging

This study validated the efficacy of the formula CL = 0.36 × 0I − 0.67 × 0T − 0.69 × T1S model on X-ray and MRI. The results showed a significant correlation between predictive value and observed value on X-ray and MRI. Specifically, the correlation coefficient (*r*) on X-ray was -0.862 with an R2 value of 0.744; on MRI, the correlation coefficient (*r*) was -0.783 with an R2 value of 0.614 (Fig. 4).

## Discussion

In this study, the sagittal plane parameters of the cervical spine measured by X-ray and MRI showed good overall inter-observer agreement. They were also similarly accurate and reliable for determining the OI. This confirms that OI is independent and stable under the influence of external factors, which is consistent with the results of a previous study [9, 20, 21]. To maintain an overall sagittal balance of the cervical spine, an increase in the angles of C0-2 and T1S-CL is required. The results of this study showed that C2S on the X-ray was positively correlated with C0-2 and T1S-CL, while it was negatively correlated with C2-7. Furthermore, OT, C2-7, and T1S obtained from X-ray measurements had greater angles compared to those obtained from MRI, while C2S and T1S-CL had smaller angles. This difference may be due to the pressure exerted on the vertebrae by the weight of the skull during X-ray in the standing position, whereas the reaction force of the bed has an effect on the vertebrae during MRI in the supine position. This is consistent with the results of a previous study [22], which found that skull contact with the table and applying a reaction force on the neck that projected posteriorly resulted in a decrease in the angle of rotation of the first thoracic vertebra, which resulted in a smaller T1S observed on MRI.

Unlike Lee’s study [9], the correlation between the OI and the C2S, C0-2, and T1S-CL is pronounced on MRI images in our study. There are several possible explanations for this diskrepancy, which include the differences in the sagittal configuration of cervical spine between Chinese and Koreans, and the differences between MRI and EOS techniques. OI may be affected by the complex anatomy of the odontoid and the difficulty in stabilizing the odontoid tip since it rotates on its own between the anterior arch of the atlas and the transverse ligament of the atlas measurement [23, 24]. We found no significant correlation between C0-2 and OI, agrees exceptionally well with what Ames et al. have concluded in their study that smaller correlation coefficients for this parameter in close proximity to the head [25]. According to the research findings of Lee and his colleagues, other factors may contribute more to the overall alignment of the cervical spine, resulting in a moderate correlation coefficient, compared to the parameter of the odontoid process closest to the head [9]. This phenomenon may result from measurement error, anatomical differences, distinctions between imaging methods, or other unknown factors.

Previous studies have demonstrated that if the CL is not sufficient to match the patient's given T1S, the second cervical vertebra will tilt forward to increase the C2S [26]. This suggests that OT decreases when the C2S angle increases, which is identical with the findings of Lee et al. [9]. These phenomena may reflect the structural interrelationships and physiological characteristics in the sagittal plane of the cervical spine. On MRI, OT was significantly correlated with C2S, C2-7, T1S, and T1S-CL, suggesting that OT may reveal the structural and positional relationships of the overall cervical spine. Specifically, a reduced OT angle may result in a forward tilt of C2S and C2-7 and a backward tilt of T1S and T1S-CL, thereby affecting the position and structure of the overall cervical spine. This study confirmed and validated the formula proposed by Lee et al. [9], for predicting the normative CL of a given patient: CL = 0.36 × OI − 0.67 × OT − 0.69 × T1S. The formula predicts CL with a high predictive accuracy in X-ray and MRI applications. Not only does this formula possibly provide a threshold for cervical deformity, but it also implies a goal for surgical correction to reconstruct the predicted physiological cervical alignment.

This study has several limitations. First, it was a retrospective study with minor variations in the location of subjects on radiographic images, which may lead to selection bias. Second, the sample size was small and only healthy subjects were included, which may not be fully representative of the entire population. To reduce heterogeneity among participants, we only included the healthy subjects who exhibited no signs of degenerative disk changes. Some normal individuals with degenerative changes (disk height loss or osteophytes) were excluded in this study, which may also create a selection bias. Third, in our study, the accuracy of OI measurements on X-ray images might be limited because of a less clear depiction of the odontoid process on X-ray images than that on MRI images.This phenomenon may lead to a slight underestimation of ICC on X-ray in our study. Fourth, we could not further analyze some factors (gender, age, and disk height loss) that influence cervical sagittal balance. These potential confounders may exert an impact on the correlation between OI and cervical sagittal parameters, and consequently, may weaken the validity of the results. Thus the results need to be interpreted with caution.

## Conclusion

In summary, OI was verified as a constant anatomic parameter, demonstrating the necessity of a combined assessment of cervical sagittal balance by employing standing X-ray and supine MRI. The formula CL = 0.36 × OI − 0.67 × OT − 0.69 × T1S can be used to predict CL in cervical sagittal parameters. This study has great significance in clinical applications, especially for medical imaging specialists who need to be aware of the differences of measurements between standing X-ray and supine MRI when evaluating cervical sagittal balance.

## Data Availability

The data used and/or analyzed during the current study are available from the corresponding author on reasonable request.

## Abbreviations

MRI: Magnetic resonance imaging
OI: Odontoid incidence
OT: Odontoid tilt
C2S: C2 slope
T1S: T1 slope
CL: Cervical lordosis
ICC: Intraclass correlation coefficient
CI: Confidence intervals
HRQOL: Health-related quality of life

## References

[1] Abumi K, Panjabi MM, Kramer KM, Duranceau J, Oxland T, Crisco JJ (1990). Biomechanical evaluation of lumbar spinal stability after graded facetectomies. Spine (Phila Pa 1976).

[2] Corey DL, Comeau D (2014). Cervical radiculopathy. Med Clin North Am.

[3] Zhou S, Li W, Su T, Du C, Wang W, Xu F, Sun Z, Li W (2019). Does lumbar lordosis minus thoracic kyphosis predict the clinical outcome of patients with adult degenerative scoliosis?. J Orthop Surg Res.

[4] Li J, Zhang D, Shen Y (2020). Impact of cervical sagittal parameters on axial neck pain in patients with cervical kyphosis. J Orthop Surg Res.

[5] Nicholson KJ, Millhouse PW, Pflug E, Woods B, Schroeder GD, Anderson DG, Hilibrand AS, Kepler CK, Kurd MF, Rihn JA, Vaccaro A, Radcliff KE (2018). Cervical sagittal range of motion as a predictor of symptom severity in cervical spondylotic myelopathy. Spine (Phila Pa 1976).

[6] Liu S, Lafage R, Smith JS, Protopsaltis TS, Lafage VC, Challier V, Shaffrey CI, Radcliff K, Arnold PM, Chapman JR, Schwab FJ, Massicotte EM, Yoon ST, Fehlings MG, Ames CP (2015). Impact of dynamic alignment, motion, and center of rotation on myelopathy grade and regional disability in cervical spondylotic myelopathy. J Neurosurg Spine.

[7] Xu S, Liang Y, Yu G, Zhu Z, Wang K, Liu H (2020). Exploration on sagittal alignment and clinical outcomes after consecutive three-level hybrid surgery and anterior cervical diskectomy and fusion: a minimum of a 5-year follow-up. J Orthop Surg Res.

[8] Chen J, Wang J, Wei X, Guan H, Wang B, Xu H, Chen J (2020). The importance of preoperative T1 slope for determining proper postoperative C2–7 Cobb's angle in patients undergoing cervical reconstruction. J Orthop Surg Res.

[9] Lee JK, Hyun SJ, Kim KJ (2022). Odontoid incidence: a novel cervical parameter influencing cervical alignment from top to bottom. Neurospine..

[10] Machino M, Yukawa Y, Imagama S, Ito K, Katayama Y, Matsumoto T, Inoue T, Ouchida J, Tomita K, Ishiguro N, Kato F (2016). Age-related and degenerative changes in the osseous anatomy, alignment, and range of motion of the cervical spine: a comparative study of radiographic data from 1016 patients with cervical spondylotic myelopathy and 1230 asymptomatic subjects. Spine (Phila Pa 1976).

[11] Tetreault LA, Dettori JR, Wilson JR, Singh A, Nouri A, Fehlings MG, Brodt ED, Jacobs WB (2013). Systematic review of magnetic resonance imaging characteristics that affect treatment decision making and predict clinical outcome in patients with cervical spondylotic myelopathy. Spine (Phila Pa 1976).

[12] Boudreau C, Carrondo Cottin S, Ruel-Laliberté J, Mercier D, Gélinas-Phaneuf N, Paquet J (2021). Correlation of supine MRI and standing radiographs for cervical sagittal balance in myelopathy patients: a cross-sectional study. Eur Spine J.

[13] Park SM, Song KS, Park SH, Kang H, Daniel RK (2015). Does whole-spine lateral radiograph with clavicle positioning reflect the correct cervical sagittal alignment?. Eur Spine J.

[14] Jun HS, Chang IB, Song JH, Kim TH, Park MS, Kim SW, Oh JK (2014). Is it possible to evaluate the parameters of cervical sagittal alignment on cervical computed tomographic scans?. Spine (Phila Pa 1976).

[15] Paholpak P, Nazareth A, Hsieh PC, Buser Z, Wang JC (2017). Kinematic evaluation of cervical sagittal balance and thoracic inlet alignment in degenerative cervical spondylolisthesis using kinematic magnetic resonance imaging. Spine J.

[16] Song G, Kenney M, Chen YS, Zheng X, Deng Y, Chen Z, Wang SX, Gambhir SS, Dai H, Rao J (2020). Carbon-coated FeCo nanoparticles as sensitive magnetic-particle-imaging tracers with photothermal and magnetothermal properties. Nat Biomed Eng.

[17] Shrout PE, Fleiss JL (1979). Intraclass correlations: uses in assessing rater reliability. Psychol Bull.

[18] Fleiss JL (2011). Design and analysis of clinical experiments.

[19] Landis JR, Koch GG (1977). The measurement of observer agreement for categorical data. Biometrics.

[20] Cheng J, Liu P, Sun D, Ma Z, Liu J, Wang Z, Mou J (2019). Correlation of cervical and thoracic inlet sagittal parameters by MRI and radiography in patients with cervical spondylosis. Medicine (Baltimore).

[21] Xing R, Zhou G, Chen Q, Liang Y, Dong J (2017). MRI to measure cervical sagittal parameters: a comparison with plain radiographs. Arch Orthop Trauma Surg.

[22] Xu C, Shen Q, Xu J, Ma J, Ye J, Mo W (2021). Comparison of cervical sagittal parameters between radiographs and magnetic resonance images in patients with cervical spondylotic myelopathy. Global Spine J..

[23] Gehweiler D, Wähnert D, Meier N, Spruit M, Raschke MJ, Richards RG, Noser H, Kamer L (2017). Computational anatomy of the dens axis evaluated by quantitative computed tomography: implications for anterior screw fixation. J Orthop Res.

[24] Lakshmanan P, Jones A, Howes J, Lyons K (2005). CT evaluation of the pattern of odontoid fractures in the elderly–relationship to upper cervical spine osteoarthritis. Eur Spine J.

[25] Ames CP, Blondel B, Scheer JK, Schwab FJ, Le Huec JC, Massicotte EM, Patel AA, Traynelis VC, Kim HJ, Shaffrey CI, Smith JS, Lafage V (2013). Cervical radiographical alignment: comprehensive assessment techniques and potential importance in cervical myelopathy. Spine (Phila Pa 1976).

[26] Protopsaltis TS, Ramchandran S, Tishelman JC, Smith JS, Neuman BJ, Mundis GM, Lafage R, Klineberg EO, Hamilton DK, LaFage V, Gupta MC, Hart RA, Schwab FJ, Burton DC, Bess S, Shaffrey CI, Ames CP (2020). The importance of C2 slope, a singular marker of cervical deformity, correlates with patient-reported outcomes. Spine (Phila Pa 1976).

